# Climate for evidence informed health system policymaking in Cameroon and Uganda before and after the introduction of knowledge translation platforms: a structured review of governmental policy documents

**DOI:** 10.1186/1478-4505-13-2

**Published:** 2015-01-01

**Authors:** Pierre Ongolo-Zogo, John N Lavis, Goran Tomson, Nelson K Sewankambo

**Affiliations:** Health Policy and Knowledge Translation Doctoral Program, Makerere University College of Health Sciences, P.O. Box 7072, Kampala, Uganda; Department of Clinical Epidemiology and Biostatistics, McMaster University, 1280 Main Street West, CRL 209, Hamilton, Ontario L8S 4 K1 Canada; Departments of Learning, Informatics, Management, Ethics and Public Health Sciences, Karolinska Institutet, Tomtebodavägen 18A, 171 76 Stockholm, Sweden; Centre for Development of Best Practices in Health, Central Hospital Yaoundé, University of Yaoundé, Yaoundé, P.O. Box 87, Cameroon

**Keywords:** Cameroon, Climate for evidence informed health system policymaking, Content analysis, Governance, Health systems, Knowledge translation platform, Low- and middle-income countries, Policy sciences analytical framework, Structured documentary review, Uganda

## Abstract

**Background:**

There is a scarcity of empirical data on African country climates for evidence-informed health system policymaking (EIHSP) to backup the longstanding reputation that research evidence is not valued enough by health policymakers as an information input.

Herein, we assess whether and how changes have occurred in the climate for EIHSP before and after the establishment of two Knowledge Translation Platforms housed in government institutions in Cameroon and Uganda since 2006.

**Methods:**

We merged content analysis techniques and policy sciences analytical frameworks to guide this structured review of governmental policy documents geared at achieving health Millennium Development Goals. We combined i) a quantitative exploration of the usage statistics of research-related words and constructs, citations of types of evidence, and budgets allocated to research-related activities; and (ii) an interpretive exploration using a deductive thematic analysis approach to uncover changes in the institutions, interests, ideas, and external factors displaying the country climate for EIHSP. Descriptive statistics compared quantitative data across countries during the periods 2001–2006 and 2007–2012.

**Results:**

We reviewed 54 documents, including 33 grants approved by global health initiatives. The usage statistics of research-related words and constructs showed an increase over time across countries. Varied forms of data, information, or research were instrumentally used to describe the burden and determinants of poverty and health conditions. The use of evidence syntheses to frame poverty and health problems, select strategies, or forecast the expected outcomes has remained sparse over time and across countries. The budgets for research increased over time from 28.496 to 95.467 million Euros (335%) in Cameroon and 38.064 to 58.884 million US dollars (155%) in Uganda, with most resources allocated to health sector performance monitoring and evaluation. The consistent naming of elements pertaining to the climate for EIHSP features the greater influence of external donors through policy transfer.

**Conclusions:**

This structured review of governmental policy documents illustrates the nascent conducive climate for EIHSP in Cameroon and Uganda and the persistent undervalue of evidence syntheses. Global and national health stakeholders should raise the profile of evidence syntheses (e.g., systematic reviews) as an information input when shaping policies and programmes.

**Electronic supplementary material:**

The online version of this article (doi:10.1186/1478-4505-13-2) contains supplementary material, which is available to authorized users.

## Background

The success of national development efforts depends upon the degree to which planners transparently use research evidence for decision making [1, 2]. The efforts initiated after the Mexico Ministerial Summit for Health Research in 2004 [3, 4] yielded achievements in terms of funding opportunities for health policy and systems research [5], knowledge translation platforms (KTPs), such as the Evidence Informed Policy Networks (EVIPNet) [6–9], and the growing knowledge base for evidence-informed health system policymaking (EIHSP). The latter is exemplified by the conceptual framework developed by the Alliance for Health Policy and Systems Research [10] and resources for EIHSP including efforts to develop guidance about health systems interventions [11–13]. The multi-faceted efforts to enable EIHSP in low- and middle-income countries (LMICs) and bridge the “know-do” gap that largely explains the predicted failure to achieve Millennium Development Goals (MDGs) 4 and 5 by 2015 in sub-Saharan countries are yet to demonstrate their effectiveness. The 2013 World Health Report has reemphasized the need to foster evidence-informed health policies, programmes, and strategies in LMICs in order to accelerate efforts towards universal health coverage [8].

**Figure 1:**
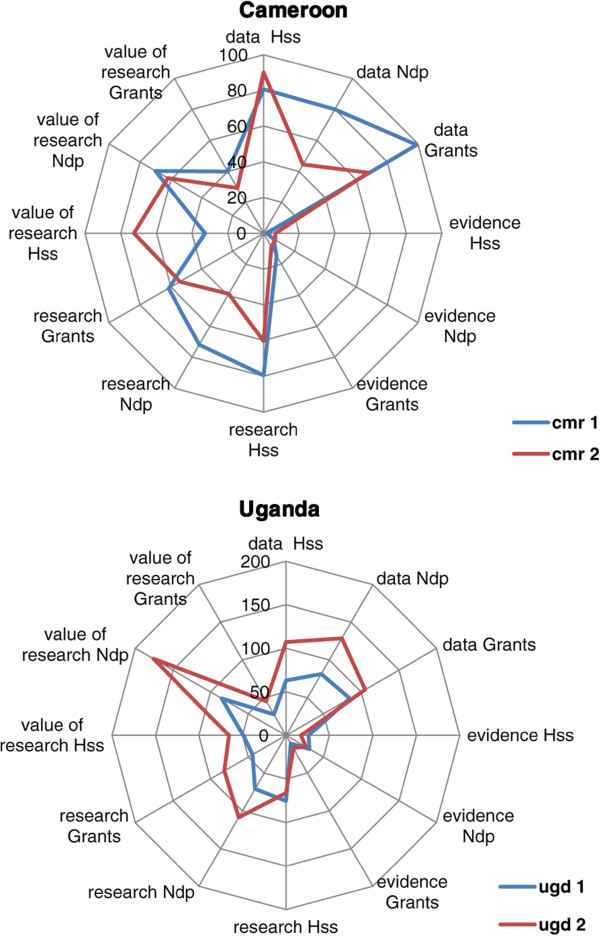
**Utilization patterns of research-related clusters in Cameroon and Uganda according to types of documents.** Ndp: National development plans, Hss: Health sector strategic plans; ugd: Uganda; cmr: Cameroon; 1: Period 2001–2006; 2: Period 2007–2012.

In that regard, there is a longstanding reputation that policymaking in sub-Saharan Africa is opaque due to factors such as governance uncertainties in contexts synonymous with scarcities, precarious democratic institutions, lack of autonomy for decision-makers [1, 2], and the neglect by the administrative elites of the invaluable contribution that research evidence can make to policy and program development [14, 15]. Furthermore, it is commonly said that research evidence is not valued enough by policymakers as an information input [16, 17]. KTPs – knowledge brokering enterprises bolstering the integrated model for linking research to policy [18–20] – are now operational in 12 sub-Saharan countries as partnerships among health stakeholders (policymakers, researchers, civil society, and media) to promote the systematic use of research evidence in policymaking about health systems through the production and dissemination of targeted evidence syntheses, the organization of evidence- informed deliberations on health priorities, and capacity building of stakeholders [9, 13, 21]. One expected outcome of these initiatives is to bring about change in the country climate for EIHSP. The latter has been defined as the range of national contextual features pertaining to the integration of research evidence in making decisions about health system policy and management. It features whether and how the social and health policy agendas and the health system actors (especially funders and research users) value the use of research evidence to inform decision-making in terms of action proposals and allocation of financial resources for evidence production and/or filtering [10, 17].

Empirical evidence on a country’s efforts to link research to policy is scarce, especially in terms of country climate for EIHSP in LMICs [10]. Scholarly efforts on the topic as from 2008 have concentrated either on tools and methods assessing the individual and organizational capabilities to produce, filter, or use policy-relevant research or on capturing stakeholders’ views, preferences, and practices [1, 11, 21–25]. Oliver et al. [26] provided a synthesis of the growing knowledge base on the technical barriers and facilitators influencing the timely use of evidence in health policymaking. In addition, Liverani et al. [27] noted the entrenched political and institutional influences on the utilization of research evidence to inform decision-making about health systems and the knowledge base was judged superficial and piecemeal. Overall, the available literature exhibits the intricacy of the prevailing political systems and institutional mechanisms with the organizational and individual resources. It is therefore critical for those striving to strengthen the research-to-policy links in a given country to assess the prevailing climate for EIHSP in order to adjust their efforts [10] and to allow a proper evaluation of the impact and influence of the multifaceted efforts being deployed by dozens of KTPs in LMICs [28, 29].

Are the KTPs established since 2006 in Cameroon and Uganda yielding any influence on the country climate for EIHSP? Our purpose was to assess whether and how changes have occurred in the country climate for EIHSP during the periods 2001–2006 and 2007–2012 in order to investigate two 6-year periods before and after the launching of both initiatives housed in government-owned institutions in Cameroon and Uganda through a structured review of governmental policy documents merging content analysis techniques with policy sciences analytical framework.

## Methods

### Theoretical underpinnings

Our approach was informed by the conceptual framework for EIHSP developed by the Alliance for Health Policy and Systems Research in its flagship report *Sound Choices, Enhancing Capacity for Evidence-Informed Health Policy* [10]. Four functions, six types of organizations, and three categories of resources are constitutive of the EIHSP infrastructure. The latter is embedded within the national context whose features are amongst others the political and governance systems, the economic and social conditions, the decision and research cultures, and regulations and legislations. The wider enabling environment is made of external funders, research institutions, and advocacy organizations and the subsequent technical capacity for health policy and systems research. Acknowledging that the EIHSP infrastructure is vulnerable to the prevailing institutions, interests, ideas, and external factors, ability to use evidence, and personal experience and intuition, we adapted the 3I + E framework – Institutions, Interests, Ideas and External factors – as the policy sciences analytical framework [13, 30] to guide our interpretive assessment of changes of the national research–health policy interfaces. Building on the collective knowledge of the authors gained from their implication (POZ, NKS) as leaders of KTP secretariats, thus investigating from within in collaboration with colleagues with major global experience of evidence to policy processes (JNL, GT) involved as co-investigators in KTP research and evaluation, we posited that the appraisal of the governance patterns and the assessment of the commitments to take action on and budget allocations for research-related activities, the use of research-related words and constructs, and the citations of research in governmental policy documents are suggestive of the country climate for EIHSP.

### Design

This structured review of governmental policy documents was guided by content analysis techniques [31] merged with a policy sciences analytical framework [13, 30]. Other scholars have used a similar approach to discern policy-specific trends from media reports [32] or from governmental policy documents [33–35]. Specifically, we have used a concurrent exploratory mixed-methods approach [36] comprising i) a quantitative exploration of the usage statistics of research-related words and constructs, and the citation of types of evidence and budgets allocated for research-related activities, and ii) an interpretive exploration of the context, the value of research, and the action proposals for EIHSP using a deductive thematic analysis to uncover the institutions, interests, ideas, and external factors displayed in the documents.

### Study geographic context

We purposively selected Cameroon and Uganda because, since 2006, they have established two pioneering KTPs in Africa (EVIPNet Cameroon housed at the Central Hospital of Yaoundé, a teaching hospital closely linked to the ministry of health, and Regional East African Community Health Policy Initiative (REACH-PI) Uganda, housed at Makerere University College of Health Sciences in Kampala, a public university) with track records of establishing vibrant partnerships of stakeholders around priority topics related to the health MDGs [9, 21]. In addition, Cameroon and Uganda feature similarities in terms of i) stable presidential regimes strongly anchored in traditional chieftaincies of 220 and 56 ethnic groups, respectively, in which the same Head of State has been in office since the 1980’s and the Parliaments are largely dominated by the Head of State’s party, and technocrats play a pivotal role during health system policymaking; ii) economic development level, as they rank as lower-middle and low-income countries, respectively, and have aligned the thrust of their development policies and plans to achieve the health MDG targets, thus becoming eligible for several Global Health Initiative (GHI) grants; iii) tiered, mixed, and poorly regulated health systems facing the dual burden of communicable diseases and rising chronic conditions with the health district as the operational unit in which state owned health services compete with private health facilities under the authority of a ministry of health coexisting with several inter-sectoral governing bodies for priority health programs; and iv) commitments to strengthen their national health research systems through established central administrative bodies in charge of health research.

### Data collection

We selected governmental development policy documents, namely a poverty reduction strategic paper (PRSP), a poverty eradication action plan (PEAP), a growth and employment strategic paper (GESP), and a national development plan (NDP), together with their subsequent health sector strategic and disease-specific strategic plans to achieve the health MDGs; the reason being that they have become the thrust of governmental visions for development since 2001 in most African countries. In addition to embodying altogether the consensus on poverty and development features, the medium- and long-term visions, and the action proposals towards growth and prosperity, these documents constitute enduring frames and anchors on which the political and administrative elites are held accountable for by the citizens. The launch of GHIs offering critical funding opportunities to accelerate the efforts towards the achievement of health MDGs has transformed the grant application processes into key markers of governmental health planning and programming. We posited that the analysis of funded grants will elucidate the national-global interplay to achieve health MDGs, especially the short- and medium-term action proposals and budget allocations for research-related activities. The analysis of these three types of governmental policy documents was judged to allow the investigation of both macro-level distributive and meso-level implementation policies [33].

We searched the government websites (Prime Minister Offices and Ministries of Health) and the websites of the GHI supporting national efforts to achieve health MDGs: Global Alliance for Vaccines and Immunization (GAVI), Global Fund to Fight AIDS Tuberculosis and Malaria (GFATM), United Nations Fund for Population (UNFPA), United Nations Child Emergency Fund (UNICEF), and the World Bank Health and Nutrition Program, to identify the funded grants to Cameroon and Uganda between January 2001 and December 2012. We checked the exhaustiveness of our search strategy with key informants from the respective ministries of health. For the quantitative analysis of usage patterns of research-related words and constructs, only the documents in PDF or Word formats were selected.

### Coding procedures

An initial round of reading of the documents provided us with an understanding of their overall structure and inspired the development of a list of research-related words or constructs. Research was broadly defined to include information generated by the health sector (e.g., management information and monitoring and evaluation (M&E) of system performance) or research conducted within the health sector (e.g., operations research) and research evidence conducted outside the jurisdiction (e.g., systematic reviews). We subsequently developed a list of research-related words and constructs in four clusters after several discussions and iterations among the authors to ensure its content validity (Additional file 1: Panel 1). The review included the following four steps for each strand:

### Quantitative data analysis

(i) The words and constructs in Additional file 1: Panel 1 were systematically searched and counted using the N*vivo software (QSR 9) and the total count for each cluster were calculated for each type of document.
(ii) The reference list of each document was examined to count and categorize the citations (A: administrative reports – annual reports of activities, inspection, and supervision reports; B: single studies – surveys and evaluation reports; C: evidence syntheses – systematic reviews, guidelines, policy briefs, evidence briefs) and we searched for any instrumental use of evidence syntheses in the document sections (e.g., problem definition or framing, strategies, implementation considerations) and their purpose with regard to the taxonomy of health systems interventions (governance, financial, and delivery arrangements and technologies).
(iii) The budget sections of the disease-specific strategic plans and funded grants were searched to record budget allocation for research-related activities (e.g., health management information, M&E, operations research, disseminating information and research, synthesizing information, research synthesis, supporting its use).
(iv) The descriptive statistics were used for the distribution of counts for each cluster of research-related words and constructs and types of citations per document as well as the financial resources allocation with an analysis of variance using the F-test to compare figures across the two periods 2001–2006 and 2007–2012, countries, and types of governmental documents.

### Qualitative data analysis

(i) The method section of each document was reviewed using a deductive thematic analysis approach to account for its development surroundings, namely timelines, stakeholders involved, and processes enacted such as public consultations, validation meetings, and guidance for preparing the document. In addition, we developed sets of key words and constructs pertaining to health system policy and management following the initial reading round of the documents.
(ii) The national development policy documents (PRSP, PEAP, GESP, and NDP) were explored with the N*vivo software (QSR 9) to search the following key words – decision making, governance, management, policymaking, and research – within their textual context (two sentences before and after the key word meaning the closest 30 to 40 words) in order to investigate the evidence-to-policy link in the following domains of social policy – education, gender, health, population, and social security.
(iii) The health sector strategic plans and disease-specific strategic plans were explored to search the following key words – decision making, evaluation, evidence, guideline, practice, quality, research, systematic review – within their textual context (two sentences before and after the key word meaning the closest 30 to 40 words) in order to investigate the evidence-to-policy link.
(iv) The grants were reviewed to search the following key words – evaluation, evidence, guideline, management, practice, quality, research, systematic review – within their textual context (two sentences before and after the key word meaning the closest 30 to 40 words) in order to investigate the features of the evidence-to-practice link.

### Qualitative interpretation of changes and explanatory factors

We used the constant comparison approach to identify changes in the institutions, interests, ideas, and external factors within each country across the two periods 2001–2006 and 2007–2012, before and after the introduction of both KTPs in 2006. Inspired by scholarly work on determinants of change in health systems and public policy [13, 30], we iteratively searched for narrative changes in governmental policy documents across the two periods in terms of diagnosis and action proposals pertaining to governance and EIHSP. We focused on institutions (e.g., policy legacies, rules of the game for public management), interests (e.g., societal interests groups), ideas (e.g., cultural beliefs, research evidence, views about what ‘ought to be’), and external factors (e.g., global development agenda, release of major reports).

### Ethics

This structured documentary review is part of a larger study that was approved by the higher degrees research and ethics committee at Makerere University, College of Health Sciences, and the Ministry of Health in Cameroon.

## Results

### Documents

In total, the search strategy identified 54 documents, namely four national development policy documents, 17 health sector and disease-specific strategic plans, and 33 grants funded by GHIs (Table 1) representing all the national development plans, the health sector strategic plans, and funded GHI grants to achieve the health MDGs produced by the governments of Cameroon and Uganda during the period 2001–2012.

**Table 1:** **List of governmental policy documents reviewed**

Year	Cameroon	Uganda
**National development plans (n = 4)**		
2003	Poverty reduction strategic paper 2003–2010	Poverty eradication action plan 2004–2008
2009	Growth and employment strategic paper 2010–2020	National development plan 2010–2015
**Health policy and strategic plans (n = 17)**		
2001	Health sector strategic plan 2001–2015	
2004		Health sector strategic plan II 2005–2010
2005	HIV-AIDS control strategic plan 2006–2010	Malaria control strategic plan 2005–2010
2006	Country multiyear immunization plan 2007–2011	Country multiyear immunization plan 2007–2011
	Malaria control strategic plan 2007–2010	HIV-AIDS control strategic plan 2007–2012
	Roadmap for reducing maternal mortality	
2007		Roadmap for reducing maternal mortality
2009	Revised health sector strategic plan 2001–2015	Health sector strategic and investment plan 2010–2015
2010	Health development plan 2011–2013	Second national health policy
	HIV AIDS control strategic plan 2011–2015	
	Malaria control strategic plan 2011–2015	
2011	Country multiyear immunization plan 2011–2015	Country multiyear immunization plan 2011–2015
**Grants (33)**		
2002	UNFPA country plan of action	Global Fund round 02 HIV-AIDS
		Global Fund round 02 Tuberculosis
2003	Global Fund round 03 HIV-AIDS	Global Fund round 03 HIV-AIDS
2004	Global Fund round 04 HIV-AIDS	Global Fund round 04 Malaria
2005	Global Fund round 05 HIV-AIDS	Global Fund round 05 HIV-AIDS
	Global Fund round 05 Malaria	
2006	GAVI health system strengthening	Global Fund round 06 Tuberculosis
2007	GAVI new vaccine support Hib	GAVI health system strengthening
	UNFPA country plan of action	Global Fund round 07 HIV-AIDS
	UNICEF country plan of action	Global Fund round 07 Malaria
2008	GAVI new vaccine support Pneumococcal	
	WBPAD health system strengthening	
2009	Global Fund round 09 Tuberculosis	UNICEF country plan of action
	Global Fund round 09 Malaria	UNFPA country plan of action
2010	Global Fund round 10 HIV-AIDS	Global Fund round 10 HIV-AIDS
	UNDAF	Global Fund round 10 Malaria
		WBPAD health system strengthening
		UNDAF
2011	GAVI new vaccine support Rotavirus	GAVI new vaccine support Pneumococcal

GAVI, Global Alliance for Vaccines and Immunization;Hib, Haemophilus influenza bacteria UNDAF, UN Development Assistance Framework; UNFPA, WBPAD, World Bank Program Assessment Document.

### Utilization patterns of clusters of research-related words and constructs

Table 2 features the counts of each cluster of research-related words and constructs and the average count per clusters and per type of document. The F-test confirms that the differences in average counts were statistically significant between the two periods in Uganda (*P* = 0.045), with higher count during the period 2007–2012. In comparing countries, the count was higher in Uganda than Cameroon in the second period (*P* = 0.07). The difference was not statistically significant between the two periods in Cameroon and between Cameroon and Uganda during the first period. Figure 1 illustrates the variations of utilization patterns across countries and periods. The cluster of words related to evidence was poorly used and remained stable across countries over time. An increase was observed in all documents in the use of data and research and the value of research in Uganda, while there was a decrease in the use of data and research in Cameroon and the value of research had increased in health sector strategic plans.

**Table 2:** **Utilization of research-related clusters according to the types of documents and periods**

	Data		Evidence		Research		Value of research	
Period	1	2	1	2	1	2	1	2
**Total count**								
Health sector and disease-specific strategic plans								
**Cameroon**	322	450	7	33	318	302	131	363
**Uganda**	189	536	79	88	226	331	147	327
National development plans								
**Cameroon**	160	89	13	15	144	78	140	124
**Uganda**	163	257	63	53	142	217	170	352
Grants								
**Cameroon**	396	679	58	88	246	541	160	293
**Uganda**	764	1.052	98	168	398	817	251	455
**Average per document**								
Health sector and disease-specific strategic plans								
**Cameroon**	81	90	2	7	80	60	33	73
**Uganda**	63	107	26	18	75	66	49	65
National development plans								
**Cameroon**	80	44	6	8	72	39	70	62
**Uganda**	82	129	31	26	71	108	85	176
Grants								
**Cameroon**	99	68	15	9	61	54	40	29
**Uganda**	85	105	11	17	44	82	28	45

1: period 2001-2006; 2: period 2007-2012.

### Utilization patterns of references

Table 3 provides details on citations. The total counts of citations were higher during the second period, although the differences were not statistically significant. The documents prepared during the period 2001–2006 were not scientifically referenced as opposed to the period 2007–2012. The analysis of the reference lists of development policy documents and health sector strategic plans showed very little use of evidence syntheses in both countries. Those cited were guidelines issued by the World Health Organization and discussion papers commissioned by international financial institutions (IFIs). A large majority of citations were single studies and survey reports. Research was instrumentally used to describe the burden and the determinants of poverty in development policy documents. In health sector strategic plans, performance monitoring data and surveys featured the burden of diseases and health inequities. There was almost no citation of systematic reviews to frame health problems or to justify the strategies selected to address the problems except in the strategic paper to reduce maternal mortality in Uganda and validated in 2007. None of the evidence syntheses relate to health system arrangements. Equally, the targets set were not justified.

**Table 3:** **Patterns of citations in national development plans and health sector strategic plans**

Documents	Administrative reports		Single studies		Evidence syntheses		Total count		Average per document	
Period	1	2	1	2	1	2	1	2	1	2
**Health sector and disease-specific strategic plans**										
**Cameroon**	5	30	25	80	0	2	30	112	15	22
**Uganda**	25	51	17	124	0	7	42	182	14	45
**National development plans**										
**Cameroon**	1	32	12	22	0	0	13	54	13	54
**Uganda**	0	48	18	47	0	0	18	95	18	98

1: period 2001-2006; 2: period 2007-2012.

### Budgets allocated for research-related activities

Table 4 outlines the budgets allocated for research-related activities in disease-specific strategic plans and grants. Most of the budgets were ear-marked for health sector performance M&E and strengthening of the health management information systems. In few cases, financial resources were allocated for conducting surveys and s0ingle studies and, in lesser cases, for systematic reviews, evidence syntheses, or guidelines. Overall budgets for research-related activities in Cameroon and Uganda during the periods 2001–2006 and 2007–2012 were, respectively, 28.496 and 95.467 million Euros (+335%) and 38.064 and 58.884 million US dollars (+155%). Hypothesizing an average exchange rate of 1 Euro = 1.2 US dollar, the budgets per capita were higher in Cameroon over time: 1.88 to 5.86 versus 1.44 to 1.80 US dollars.

**Table 4:** **Financial resources for research activities in disease-specific strategic plans and grants**

Year	Cameroon	Activities	Budget (€)	Uganda	Activities	Budget ($)
**Health strategic plans**						
2005	HIV AIDS	Surveillance + research	8,462,451	Malaria	Research + M&E	5,251,755
2006	Immunization	N/A	N/A	Immunization	N/A	N/A
	Malaria	Surveillance + research	13,594,900	HIV AIDS	Research + M&E	18,531,500
	Maternal mortality	Research	61,068			
**Total 2001-2006**			**22,118,419**			**23,783,255**
2007				Maternal mortality	Research + M&E	544,573
2010	HIV AIDS	Strategic information + research	38,085,245			
	Malaria	OR + M&E	28,981,636			
2011	Immunization	N/A	N/A	Immunization	N/A	N/A
**Total 2007-2012**			**67,066,881**			**544,573**
**Grants**						
2002	UNFPA	N/A	N/A	GFR02 HA	Data + IS	5,534,446
				GFR02 ML + TB	M&E	1,501,000
2003	GFR03 HA + ML + TB	M&E + OR	3,485,615	GFR03 HA	Research + M&E	1,800,000
2004	GFR04 HA	N/A	N/A	GFR04 ML	M&E	1,493,150
2005	GFR05 HA	M&E	1,107,281			
	GFR05 ML	OR + M&E	677,620			
2006	GAVI HSS	M&E + studies + survey	1,107,177	GFR06 HA + TB	M&E	3,952,429
**Total 2001-2006**			**6,377,693**			**14,281,025**
2007	GAVI NVS HIB	Post introduction evaluation	22,674	GAVI HSS	Surveillance + M&E	250,000
	UNDAF (UNFPA+ UNICEF)	IS on social issues + surveys and census	1,278,625	GFR07 HA	M&E	15,106,658
				GFR07 ML	IS + M&E	1,867,234
2008	GAVI NVS Pneumococcal	Post introduction evaluation	22,900			
2009	GFR09 TB	OR + M&E	1,694,208	UNICEF	IS + evaluation	6,857,000
	GFR09 ML	IS + M&E	20,621,583	UNFPA	Data + IS	7,000,000
2010	GFR10 HA	IS + M&E + generation of strategic information	4,703,780	GFR10 HA	IS	5,439,679
				GFR10 ML	IS + M&E	21,743,885
				UNDAF	N/A	N/A
2011	GAVI NVS Rotavirus	M&E	57,252	GAVI NVS Pneumococcal	Surveillance + M&E	75,000
**Total 2007-2012**			**28,401,022**			**58,339,456**

IS, Information systems; OR, Operations research; M&E, Monitoring and evaluation; N/A, Not available; GFR, Global Fund Round; HA, HIV-Aids; ML, Malaria; TB, Tuberculosis; HSS, Health systems strengthening; NVS, New vaccines support; GAVI, Global Alliance for Vaccines and Immunization; UNDAF, UN Development Assistance Framework.

### The context in which the governmental policy documents were developed

The political systems have remained stable with the same leading majority in both countries during the 12-year period in spite of several presidential and legislative elections. The poverty level has remained above 25% of the population in both countries in spite of the improving overall economic conditions. The development policy documents were prepared under the guidance of the World Bank and the International Monetary Fund as part of their post structural adjustment measures under the Enhanced Initiative for Heavily Indebted Poor Countries (HIPC Initiative) and the subsequent Poverty Reduction and Growth Facility (PRGF). The main objective of these plans was to reduce poverty through a strong and sustainable economic growth, an increased efficiency of public expenditure and effective targeting of poverty reduction policies, and enhanced governance geared to attain the MDGs. Bilateral and multilateral donor agencies were also involved in both countries.

The formulation phases were marked in both countries and over time by nationwide iterative, inclusive, and participatory processes bringing together the population at the grassroots, donor agencies and development partners, public administration and government officials from different levels, civil society organizations and private sector, academicians and consultants, both national and international. There were combinations of top-down and bottom-up approaches, including participatory diagnosis and priority setting steered by a multi-sectoral body equipped with a technical secretariat to draft the plan. The IFIs provided guidance and contributed resources for the participatory processes in addition to recommending the appropriate macroeconomic analytical and decision frameworks.

The preparatory phase of the first generation of development policy documents displayed policy transfer and learning experiences on how to engage politicians and bureaucrats with representatives from non-state sector into fair participatory and transparent processes for framing poverty reduction priorities and strategies. It took almost 2 years in both countries to complete the formulation stage – reaching the final version signed by the Prime Minister in Cameroon and the President of the Republic in Uganda. The IFIs and development partners supported several analytical works such as comprehensive studies on the sources of growth, analyses of poverty dynamics, and modelling work to align the priority medium term expenditure frameworks with the macroeconomic and budgetary frameworks. Both countries were challenged to align the poverty reduction strategies and the needs expressed during the participatory consultations. The formulation of the second generation of development policy documents was shortened by the multi-sectoral advisory and consultative frameworks established to monitor the implementation of the first generation plans. Preparatory work included activities such as review of sector strategies, household surveys, and studies on progress towards MDGs, and mapping of macroeconomic and budgetary guidelines through medium-term budgetary frameworks. The review and evaluation of the first generation plans (PRSP and PEAP) took place in the midst of the 2008 international financial crisis and national social crisis against rising costs of food products. The PRSP was replaced by the GESP in the case of Cameroon and the PEAP was replaced by the NDP in the case of Uganda. It was deemed necessary to formulate a longer term economic development vision. The GESP and NDP were aligned to the 2005 Paris Declaration on the effectiveness of official development assistance. These plans henceforth served as the reference framework of government policy and actions and the point of convergence for development partners. The period 2007–2012 was marked by the institution of the United Nations Development Assistance Framework (UNDAF). Uganda experienced donors’ alignment efforts during both periods under a sector wide approach (SWAp), as opposed to Cameroon, where negotiations for a health SWAp were initiated in 2005 and still pending during the second period.

All the health sector strategic and disease-specific plans were prepared with guidance and support from WHO, UNICEF, and UNFPA, and their formulation phases combined top-down and bottom-up participatory approaches with a central technical secretariat. Stakeholders involved included government officials, civil society representatives, private healthcare providers, and academicians. Guiding principles included responsiveness to population needs, performance, quality of care, and equity.

### Institutions displaying the climate for EIHSP

Tensions were described around the irreversible turn towards the market economy and the consequences of the structural adjustment plans imposed by the IFIs during the 1990’s. The private sector was endowed with a critical role in combating poverty and ensuring prosperity in both countries over time. The salient features of governance systems as described in documents were i) the numerous weaknesses of the public administration and management, ii) the pervasive corruption evidenced in several reports of indices of corruption perceptions by Transparency International and the “Doing Business” reports by the World Bank, iii) the challenged implementation of the decentralization enshrined in both Constitutions, and iv) the inappropriate access to information due to the poor information and communication technologies infrastructure and the under-resourced media sector (Table 5).

**Table 5:** **Selected quotations diagnosing the climate for EIHSP**

Elements	Cameroon 2001–2006	Cameroon 2007–2012	Uganda 2001–2006	Uganda 2007–2012
**Institutions**	✓Corruption and poor public management ranked as important determinants of poverty	✓Some kind of slowness continues to hamper the implementation of decentralization and finds justification in the late operationalization of national council on decentralization and the inter-ministerial committee on local services	✓Perceptions about corruption remain a concern	✓Corruption is most rife in procurement, administration of public expenditure, and management of revenue
			✓The major challenges to M&E include weak coordination arrangements, parallel M&E efforts, poor public management culture, gaps in information, and underused information	
**Interests**				✓Lack of ethics integrity and patriotism and a tolerance for corruption
				✓Citizenry is not fully empowered to engage effectively in demanding better performance from Government institutions in meeting their obligations and providing services
**Ideas**			✓Discrimination against women through traditional rules and practices that explicitly exclude them or give preference to men is a key constraint on women’s empowerment and economic progress	✓Traditions, culture, and religious norms are not supportive to modern approaches in society and have, therefore, limited economic growth and structural transformation. Backward cultural practices, beliefs, attitudes, and a lack of national ethical values in political, social, and economic spheres

M&E, Monitoring and evaluation.

In development policy documents, most action proposals during both periods focused on fixing the administration and public management weaknesses towards the principles of “New Public Management” fostering results-oriented management and fiscal and budgetary reforms (Table 6). The second pillar of reforms pertained to improving governance with a special attention to increasing public participation, enhancing accountability, curbing corruption, reinforcing the involvement of the civil society, and the private sector in public policymaking. Social public policies were ranked in third position after the “so called” growth sectors and prominence was given to addressing gender-based inequalities, social inequities, and improving access to education and health services. Enhancing access to information was planned through the privatization of information and communication technologies services in both countries (Table 6).

**Table 6:** **Selected quotations of action proposals related to the climate for EIHSP**

Elements	Cameroon 2001–2006	Cameroon 2007–2012	Uganda 2001–2006	Uganda 2007–2012
**Institutions**	✓Increase inclusive and participatory M&E of poverty reduction projects	✓Strengthen the entire public policy planning, programming, budgeting, and monitoring process	✓Reform the public sector to strengthen performance management through results-oriented management	✓Develop guidelines for policy initiation and formulation
	✓Bring together civil society and development partners to discuss major directions in economic and social policies as well as in development strategy management			✓Ensure that policies are based on sound research, analysis, and evaluation
		✓Deepen the decentralization process		✓Strengthen decentralization
	✓Define and organize analytical work programs	✓Introduce a results-oriented M&E system		✓Enhance transparency and accountability
	✓Strengthen capacities to guide development management, including statistical work and economic modelling work for preparing medium-term macroeconomic and sector frameworks	✓Generalize medium term expenditure frameworks and programme budgets		✓Further the current framework for involvement of the private sector and civil society in public policymaking, planning, and implementation
	✓Perform poverty and social impact analyses	✓Continue to modernize the public administration by improving the institutional, administrative management and governance framework		✓NDP will guide decision making and implementation of government programmes including the annual budget process, and the prioritization and direction of government actions
**Interests**		✓Strengthen the capacity of the various players of the economy	✓Create incentives for technological development and implement industrialisation policy to promote technical transformation	✓Address the pay reform strategy focusing on job evaluation targets of technical cadres
		✓Implement a system of incentives		✓Preserve the purchasing power of public salaries
				✓Establish and support the research fund
				✓Promote research and development, commercialization, and adoption of scientific research and patenting intellectual property
**Ideas**	✓Develop a poverty information system by upgrading the current statistical system so as to provide public agencies, private sector, development partners, and civil society with timely information required to i) effectively manage poverty reduction activities, ii) serve the needs of several poverty analysis objectives, and iii) provide timely readings of quantitative or qualitative indicators for regular analysis		✓National integrated M&E strategy to include censuses, surveys, administrative data, beneficiary assessments, and research studies	✓The NDP will first and foremost be an instrument of evidence-based political commitment used to capture the public imagination and commitment for the next phase of nation building. While the PEAP stressed poverty eradication and prioritized social services, the NDP maintains the poverty eradication vision, but with an additional emphasis on economic transformation and wealth creation thereby intertwining sustainable economic growth with poverty eradication
				✓Create a strong and responsive human resource base equipped with positive values and attitudes to generate and support accelerated growth, employment creation, and prosperity for socioeconomic transformation
			✓PEAP Results and Policy Matrix will be used to guide monitoring	✓Transform mind-sets, attitudes, cultural practices, and perceptions to appreciate productivity and development

M&E, Monitoring and evaluation; NDP, National development plan; PEAP, Poverty eradication action plan.

The decision culture within both health systems was marked by efforts to implement the decentralization according to the health district model championed by the African Regional Office of the World Health Organization. In Uganda, the Health Policy Analysis Committee (Additional file 1: Panel 2) has progressively played a prominent coordinating role while in the case of Cameroon a technical secretariat was established to steer and pilot the implementation of the health sector strategy with less responsibility. The weak public management also manifested within the health sector through inappropriate governance, pervasive petty corruption and weak community participation to decision making at the district level, ineffective health management information systems, centralized resources allocation, and top-down planning frequently overlooking the district needs. Both countries have experienced emerging multi-sectoral governing bodies for priority public health programmes such as the Inter Agencies Coordination Committee to steer the Expanded Program of Immunization or Country Coordination Mechanisms to oversee grants from the GFATM. These committees have created new spaces for the non-state actors to influence health policymaking and enhance accountability through social control mechanisms. New rules of the game were equally set for health planning and programming through mandatory external and participatory evaluations. Furthermore, the civil society organizations (CSOs) became eligible to GHI competitive grant applications. Some applications from CSOs to the Global Fund to fight AIDS, Tuberculosis, and Malaria were more successful than government applications during the period 2007–2012.

### Interests influencing the climate for EIHSP

There were no changes across periods and countries. At the forefront were the bureaucrats and health workers described as lacking work ethics and prone to corruption and migration in the private sector or abroad. The main explanatory factors were the lack of policy and regulations on conflicts of interest, the poor management of scarce human resources, and the low salaries in the public sector. Health bureaucrats and providers were confronted to competing public and private health care organizations and non-governmental organizations offering more attractive salaries than the state payroll across countries. Informal practice in state-owned health facilities were criticised in both countries with high prevalence of petty corruption. During the period 2007–2012, Uganda instituted a new pay and bonus scheme in order to retain civil servants in the priority public health programs and the hiring practices were reformed in order to ensure that senior staff and management of public service were on contract employment terms so as to reduce migration in the private sector. The shortage of human resources for health across countries led to a burst of community health worker programmes and task shifting in most primary healthcare facilities.

In both countries, the CSOs experienced a rapidly expanding role more or less entrenched in donor-dependency and confronted with unprepared regulatory frameworks. The CSOs and private healthcare providers were progressively given more salience as critical health stakeholders with the introduction of some forms of performance-based financing and contracting to supplement state-owned facilities. The eligibility of non-state actors to GHI grants has generated a new impetus for CSOs. The action proposals exhibited features of the “new public management” principles, meaning performance-based financing, results-oriented management, and greater social control of public actions by CSOs, thus fuelling some tensions around their funder dependency.

### Ideas displaying the climate for EIHSP

Manifestations and determinants of poverty as perceived by populations at the grass roots were abundantly described and poverty magnitude mainly evidenced by statistics from surveys and routine monitoring data. The infrastructure and resources to produce reliable data and information were admittedly weak and inappropriate in spite of improvements observed over time across countries in terms of capacities to analyse population and poverty data. In Uganda, traditions and cultural mind-sets were underscored to fuel gender-based inequities and the negative attitudes towards modernity and science, technology, and innovation. The low profile of science, technology, and innovation in both countries was evidenced by weak infrastructure and a low ratio of science/arts students in institutions of higher education.

All the plans exhibited action proposals related to enhancing the science, technology, and innovation sector and strengthening the reliability of the information systems to provide timely and appropriate information to monitor progress, ensure greater accountability, and to guide corrective actions with a clear emphasis on equity. The second generation plans were framed more positively by giving prominence to growth, employment, prosperity, skilled, evidence-based society, and economic emergence as compared to fighting against poverty and inequity (Table 6).

The first period was marked by efforts to resuscitate both health sectors from the hardship consecutive to structural adjustment programmes and the booming of vertical priority public health programmes to fix the enduring and widening health inequities. M&E and operations research were given salience as markers of evidence-informed decision making. Performance M&E and financial accountability of health programmes were emphasized as indicators of success. Policy transfer and learning was exhibited across countries in terms of approaches and tools for strategic planning, macroeconomics analytical frameworks, participatory diagnosis, and selection of poverty reduction strategies. Countries were provided with increasingly stringent guidance for GHI grant applications on themes such as accountability, equity, financial risk assessment, openness, performance, public participation, responsiveness, and quality. Furthermore, grant applications to GAVI and GFATM were supported by United Nations agencies as well as bilateral agencies such as the United States Agency for International Development in Uganda through the organization of workshops and the hiring of consultants to assist countries complying with stringent application forms and guidelines. Additional file 1: Panel 3 illustrates the multi-faceted support provided for a GFATM Round 7 grant application in Uganda during the second period and the same was observed in Cameroon.

### External factors playing a role in the climate for EIHSP

The remarkable factors were the impetus generated by the United Nations Millennium Summit, the African Union endorsing the MDGs, and the influence of IFIs through their post-structural adjustment measures. The HIPC Initiative and the PRGF brought forward new budgetary policies, such as Medium Term Expenditure Frameworks, fiscal and public finance reforms, and state procurement reforms, to fight corruption and improve the private business environment with a periodic international ranking of countries performance.

The HIPC Initiative introduced some forms of coercion upon governments to create new inclusive political spaces providing room for the CSOs and the private sector to contribute to public policymaking against poverty and to enhance growth and employment. The paradigm shift in the naming of national development efforts from poverty reduction/eradication to growth and employment, economic emergence, and prosperity was inspired by the IFIs. As a consequence of the Paris Declaration on the effectiveness of official development assistance, the UNDAF has been instrumental across countries during the period 2007–2012 to streamline the intersectoral coordination and alignment of developmental efforts.

The Transparency International corruption index reports and the World Bank Doing Business reports have maintained government officials under pressure to fight against corruption within the health services. Most GHI grant application forms strongly emphasized the need to strengthen the CSOs and community participation to ensure responsiveness to population needs. The setting of continental health targets by the African Union Summit in Abuja in 2006 and the institution of the UNDAF have influenced national choices in terms of priority health strategies across countries. Examples are the development by each country of a national roadmap to reduce maternal and child mortality and national immunization policies.

In the field of immunization, the “Global Immunization Vision and Strategy 2006–2015” has set the alignment frame by providing a clear division of labour among global, regional, and national efforts against the systemic barriers to vaccination in order to reinforce health systems and to achieve MDG 4. As a consequence, countries were advised to adapt their strategies to the lessons learned from implementing the regional EPI strategic plan 2001–2005 and, particularly, to incorporate the crucial factors for success (e.g., firm political commitment, micro-planning at the district level with the participation of the communities, appropriate supervision, and strong district-based monitoring systems). The division of labour assigned roles and responsibilities to countries in terms of preparing comprehensive multi-annual vaccination plans, giving priority to cooperation and multi-sector partnership arrangements, strengthening human resources for immunization, increasing the ear-marked financial resources for vaccination, and updating vaccine policies and guidelines. In the meantime, the WHO and UNICEF were to maintain high the profile of immunization and vaccines as well as to help countries in terms of advocacy, technical, financial, and material support for the priority activities, and coordination and partnership arrangements.

### Interpretive synthesis

The reputation that research evidence is not valued enough by policymakers as information input needs to be nuanced. The problem-solving approach to health policymaking, planning, and programming has introduced some degree of rationality. When comparing the periods 2001–2006 and 2007–2012, there is empirical evidence that the climate for EIHSP, as displayed in governmental policy documents, has improved in Cameroon and Uganda. The changes can be related to several factors, including some coercive policy transfer instilled by external donors and the multi-faceted global push to achieve MDGs and to foster EIHSP, which led to the establishment of EVIPNet Cameroon and REACH-PI Uganda in 2006.

The governmental policy documents prepared during the period 2001–2006 were not scientifically referenced in comparison to those issued during the period 2007–2012 with Ugandan documents being overall better referenced. One reason is the longstanding institutional arrangements for donor coordination under a SWAp and the existing health policy advisory committee established in the late 1990s. However, the justifications about how the options and strategies were selected were generally missing across countries and over time as opposed to the extensive description of the burden of poverty and health conditions. The evolving discourse on the problem-solving approach and results-oriented systems, as well as EIHSP, was displayed in both strategic papers and approved grants across countries and periods with a stronger rhetorical emphasis in Uganda (Table 6). The low profile of evidence syntheses has remained stable in both countries in terms of budget allocations and in terms of use to frame poverty and health problems. Almost none of the documents described the process and trade-offs made to select the appropriate options and strategies or to forecast the health outcomes expected from the developmental efforts.

The wider environment was marked by the impetus to alleviate poverty and aligning efforts to improve health, equity, and governance. There were several features of policy transfer, learning, and diffusion, including some forms of coercion particularly with stringent processes to diagnose poverty reduction priorities and grant application forms across countries over time. The role played by external donors and technical partners (e.g., IFIs, UNFPA, UNICEF, and WHO) has grown greater over time across countries. The alignment efforts exhibited in the second period could be related to the Paris Declaration on the effectiveness of official development assistance and the institution of one UNDAF; the high level priority given to governance, equity and gender, and science and technology are examples of such. Indeed, the same macroeconomics analytical frameworks and tools recommended by the IFIs were used in both countries to reform public management and financial sectors and the same approaches to participatory consultations to diagnose poverty and to identify priority poverty reduction strategies were applied in both countries.

The Transparency International ranking of perceptions of corruption and the Doing Business ranking in both countries were instrumentally used to establish the explicit linkages between poor governance and poverty and to justify the institution of mechanisms to improve public management performance, to fight corruption, and to strengthen the decentralization process as a means of improving public accountability. Action proposals were explicit in terms of strengthening the national statistics sector and poverty M&E mechanisms.

The guiding documents for GHI grant applications have evolved to include detailed guidelines on the expected content and the desirable preparation and validation processes. The influence of consultants for technical assistance during the development phase was obvious in both countries. Budget allocation was markedly skewed towards health system performance M&E with special attention to financial operations as opposed to linking those to health outputs and outcomes.

## Discussion

### Key findings

This structured review of 54 governmental policy documents merging content analysis techniques with policy sciences analytical frameworks contributes empirical evidence on the emerging enabling climate for EIHSP in Cameroon and Uganda and the enduring undervalue of evidence syntheses as opposed to operational research and systems performance M&E. Varied forms of data, information, or research results were instrumentally used to describe the burden and determinants of poverty as well as the burden of health conditions. Budgets allocated for research-related activities have increased over time in both countries. Sparse use of and low support for evidence syntheses have remained stable over time and across countries as illustrated by the usage statistics and budget allocations. The utilization patterns of types of evidence, the naming, and the consistency of elements featuring the climate for EIHSP suggest some influence of the socioeconomic and political contexts. The latter were marked by the pervasive weaknesses of national public management sectors and the global impetus to speed up efforts to achieve the MDGs with a strong emphasis on the imperative to improve public governance and accountability. Another prominent feature of context in both countries was the greater influence of development partners during health policymaking, planning, and programming through some forms of coercive policy transfer. Over time and across countries, civil society organizations have gained prominence in public policymaking and the enduring issues of concerns regarding the health workers were repeatedly lack of motivation and petty corruption.

### Strengths

To our knowledge, our study is the first to combine quantitative and qualitative content analysis techniques with policy sciences analytical frameworks to reduce the dearth of empirical evidence on the country climate for EIHSP in LMICs [10, 21, 37]. This is an important methodological and empirical contribution to the ongoing efforts to strengthen the knowledge base in the field of EIHSP. This longitudinal analysis over a 12-year period of the climate for EIHSP in two African countries lays the ground for further development of appropriate strategies to create and sustain enabling environments that are called for since 2004 [3–7, 10] and reemphasized by the 2013 World Health Report [8]. Furthermore, this cross-country longitudinal assessment of the prevailing governance systems and decision cultures also features the long term action proposals and budget allocations for EIHSP. This mapping of institutional structures is a tentative response to the call for critically understanding the optimal ways and emerging opportunities to strengthen EIHSP [21, 23, 24]. It also illustrates how an innovative approach using an automated text analysis tool can apply to reviewing 54 governmental policy documents. Finally, this study features several aspects of policy transfer, learning, and diffusion [38, 39] across countries and fuelled by global commitments to accelerate progress towards the MDGs [2, 14, 15].

### Limitations

This review presents several limitations. First, it could be criticised for using an automated word quantitative extraction and for restricting the textual context for qualitative analysis [30]. However, the combination of quantitative and qualitative content analysis with policy sciences analytical frameworks has enabled us to capture critical features of the climate for EIHSP. Second is the very nature of the structured review of governmental policy documents that prevents such an exercise to capture the politics that prevailed during their formulations [34, 35]. Similarly, the review of the approved grants and validated strategic papers does not allow accounting of the influences and power struggles that could have happened between national governments, civil society organizations, and external donors. By design, this study fails to elucidate the respective contribution of the individual factors (e.g., intuition, skills, and attitudes of those involved in preparing the governmental policy documents) and the contextual political factors [1, 2, 13, 14, 23–27]. We have limited the scope of the analysis to grant documents from multinational donors. By doing so, we have probably overlooked the political dynamics pertaining to bilateral cooperation and the policy impact of agencies such as the United States Agency for International Development in Uganda or the Agence Française de Développement in Cameroon. However, the review of health strategic plans implicitly takes into consideration the role of these bilateral donors. Finally, it was beyond the scope of this review to examine the actual accuracy of the citations and how much the strategies and options selected for implementation in the grants and strategic plans were evidence based, as such an exercise would require different research design and methods [33].

### Findings in relation to other studies

The integration of contextual features over 12 years, the use of evidence in governmental policy documents, the approximation of financial resources allocated to research related activities, and the examination of the institutions, interests, ideas, and external factors displaying the climate for EIHSP in Cameroon and Uganda go beyond what is known so far on the climate for research use in LMICs [8, 10, 17, 21–23]. Most studies have focused on individual and organizational dimensions of climate for using research evidence during health system policymaking. The increasing use of research-related words and constructs in governmental policy documents is coherent with the policymakers’ positive attitudes vis-à-vis research use [24, 25]. The scarcity of evidence syntheses in reference lists across countries and over time suggests individual limitations in the access to and appraise of systematic review and economic analyses as well as the discomfort of certain policymakers with such types of evidence [24, 26]. The framework to assess country efforts to link research to policy laid the groundwork for evaluating country climate by suggesting dimensions and elements for assessment [17]. Some of these elements were investigated through structured reflection with stakeholders [21] and our findings on the mechanisms for policy transfer and learning [38, 39] align strongly to elucidate the urgent need for comprehensive analysis of country context in order to tailor knowledge translation strategies. This study is an attempt to uncover features of the intricacy of politics and organizational and individual factors pertaining to the research-to-policy interface [10, 27] in two African countries. Our results suggest the currency of calls issued since 2004 for a greater support for and a sound use of evidence syntheses (e.g., systematic reviews, evidence briefs for policy, etc.) by all health stakeholders while making decisions about health system policymaking, planning, and programming [3–5, 8, 10].

### Implications for policymaking

Our results have implications for national and global health stakeholders, especially those shaping health policies, plans, and programs. The sparse use of evidence syntheses to frame health problems and to select and justify the options and strategies [40, 41] to address those problems is fuelling, at least partially, the failure to achieve the targets set as those might be overestimated [11]. The silence over the trade-offs along the selection of strategies is most likely to impede any readjustment of the strategies in the case of unexpected unsatisfactory health outcomes [11]. There are pressing needs to reinforce the capacities and skills of consultants, policy analysts, and planners in order to enhance how they value evidence syntheses and economic analyses [24] and the scientific quality of governmental policy documents. The evidence syntheses constitute a critical asset for international and national bureaucrats striving to enhance the effectiveness of social policies not only in terms of accountability and performance monitoring but to linking them with health outcomes [11, 40]. Health system policymakers need to be cognisant of the knowledge base on EIHSP or on their respective domains and the growing number of relevant evidence syntheses [41].

### Implications for researchers

Efforts towards preparing guidance for evidence-informed decision making about health systems require the developers to appropriately incorporate the contextual features that will influence the adoption and implementation of such tools at national and sub-national contexts in LMICs with varied infrastructure for EIHSP [12, 13]. The methods used in this study can inspire further applications to assessing country climate for EIHSP. Such investigations are particularly needed now [10, 37], as many constituencies are engaged in funding efforts to strengthen national health research systems in order to accelerate efforts towards universal health coverage [6–8] in addition to laying the ground for proper evaluation of the impact and influence of KTPs in countries synonymous with scarcities. Scholars investigating KTPs should include an as comprehensive as possible examination of the context in which the KTPs operate in order to not only design the most appropriate strategies but also to ably assess the impact and influence of their efforts. Integrating evidence into policy requires integrating political and institutional contexts in which policymaking occurs [26, 27, 37, 40–43].

## Conclusions

This structured review of governmental policy documents features an emerging conducive climate for EIHSP in Cameroon and Uganda favoured by a problem-solving approach to poverty reduction and health policymaking, planning, and programming and mechanisms of policy transfer instilled by external donors. The enduring low profile of evidence syntheses suggests the needs to further strengthen the capacities of stakeholders to demand for and use systematic reviews when shaping policies and programmes about health systems. The methodological approach used in this study to uncover the climate for EIHSP constitutes a sound option for scholars investigating the impact and influence of KTPs as it accounts for the intricacy of the political and institutional factors with the evidence-to-policy interface.

## Electronic supplementary material

Additional file 1: **Panel 1: clusters of research-related words and constructs.** Panel 2: The Health Policy Advisory Committee (HPAC) and the Partnership Committee (PC) in Uganda. Panel 3: Donors’ support for grant application to GFATM. (DOCX 16 KB)

## Abbreviations

CSO: Civil society organization
EIHSP: Evidence informed health systems policymaking
EVIPNet: Evidence informed policy network
GAVI: Global alliance for vaccines and immunization
GESP: Growth and employment strategic paper
GFATM: Global fund to fight AIDS, Tuberculosis and malaria
GHI: Global health initiative
HIPC Initiative: Enhanced initiative for heavily indebted poor countries
IFIs: International financial institutions
KTP: Knowledge translation platform
LMICs: Low- and middle-income countries
MDGs: Millennium development goals
M&E: Monitoring and evaluation
NDP: National development plan
PEAP: Poverty eradication action plan
PRGF: Poverty reduction and growth facility
PRSP: Poverty reduction strategic paper
REACH-PI: Regional East African community health policy initiative
SWAp: Sector wide approach
UNDAF: United nations development assistance framework.
