# Supplementary material for: Climate for evidence informed health system policymaking in Cameroon and Uganda before and after the introduction of knowledge translation platforms: a structured review of governmental policy documents
Source: Health Res Policy Syst. 2015 Jan 1;13:2. doi: 10.1186/1478-4505-13-2 (PMC4298114; doi:10.1186/1478-4505-13-2)
Supplement: Supplementary file 1 — Additional file 1: Panel 1: clusters of research-related words and constructs. Panel 2: The Health Policy Advisory Committee (HPAC) and the Partnership Committee (PC) in Uganda. Panel 3: Donors’ support for grant application to GFATM. (DOCX 16 KB) [file 12961_2014_369_MOESM1_ESM.docx]

Additional file 1

Panel 1: clusters of research-related words and constructs

| **Cluster** | **Words** |
| --- | --- |
| **Data** | Data – epidemiolog* – information – monitoring – statistics – surveillance |
| **Research** | Analysis – evaluation – research – study – survey |
| **Evidence** | Best practices – evidence – guidelines – knowledge – lessons learned – systematic review |
| **Value of research** | Equity – governance – innovation – quality – science – technology |

| - HPAC is composed of representatives of the Ministry of Health, Health Development Partners, National, Regional Referral and District Health Care Delivery levels, Health Service Commission, Uganda AIDS Commission, Private-not–for–Profit Medical Bureaus, and other Line Ministries (Public Service, Finance, Education, Water and Environment, Gender, Local Government), Civil Society Organisations, Faith based organizations, Private Sector, and people living with the diseases. Representatives of CSOs, FBOs, Private Sector and people living with the disease are selected by their own constituencies. - HPAC holds monthly meetings chaired by the Permanent Secretary of the Ministry of Health and reports to the Top Management Committee of the Ministry of Health. HPAC is supported by seven Technical Working groups (TWGs) namely Human Resources for Health; Health Infrastructure; Medicines Management and procurement; Basic Package, Sector Budget; Supervision, Monitoring and Evaluation; Public Private Partnership in Health. HPAC, as the SWAp coordination mechanism, advises government on priorities, policy implementation, and conducts regular joint reviews of health sector performance. - The Technical Review and Joint Review Missions are mechanisms for joint assessment of progress of health programmes implementation. Decision making is by consensus and in cases of disagreements they are resolved by open voting and in that case the Ministry of Health carries one vote |
| --- |
| - The PC comprises representatives from 12 constituencies that make up the Uganda HIV/AIDS Partnership: National Parliament, Central Government Ministries, Local Governments, AIDS Development Partners, People living with HIV/AIDS networks and organizations, Private Sector, International and National Non Governmental Organizations, Faith-Based Organizations, Young People, Research Academia and Science, Media arts and Culture. - The PC serves as a forum for oversight and coordination of the HIV/AIDS national response, planning, decision-making on national priorities, resources mobilization, as well as monitoring and evaluation. PC coordinates its functions with the Uganda AIDS Commission, which serves as its secretariat and the Parliamentary committee on HIV/AIDS and the Parliamentary Committee on Social Services. In Uganda, most organizations, networks and associations of Persons Living with HIV/AIDS (PHAs) are coordinated by a nationally registered institution known as National Forum of People Living with HIV/AIDS (NAFOPHANU). PHA organizations and networks are recognized as a distinct constituency under the National AIDS Partnership with representation to PC. The PHA constituency has two representatives to the PC, has representation on all sub-committees of the PC and is actively engaged in all activities of the Civil Society Inter-constituency Coordination Committee (CICC). CICC provides civil society with a forum to promote inter-constituency coordination, and to ensure harmonization and alignment. |

**Panel 2: The Health Policy Advisory Committee (HPAC) and the Partnership Committee (PC) in Uganda**

| **Donor** | **Types of support** | **Duration (weeks)** |
| --- | --- | --- |
| **USAID** | Institutional arrangements for Global Fund and finalizing terms of Partnership Committee operations manual | 2 |
| **WHO** | Technical and financial for drafting the proposal: attendance of workshop on capacity building for proposal development in Harare Zimbabwe. | 3 |
| **UNAIDS** | Facilitation of 2 writing retreats for the drafting team | 2 |
| **WHO** | Proposal development process of the Tuberculosis component | 5 |
| **UN Millennium Project** | Malaria component Formulation of proposal draft zero | 2 |
| **WHO-AFRO, Inter-country Support Team for East and Southern Africa** | Malaria component Facilitating the processes of inclusiveness, budgeting and work plan preparation | 4 |
| **WHO, Uganda Country Office** | Malaria component Proposal writing and facilitating retreats during the writing process | 10 |

**Panel 3:** **Donors’ support for grant application to GFATM**
